# Corrosion of food grinding discs in gastro-intestinal environment

**DOI:** 10.1016/j.heliyon.2023.e20523

**Published:** 2023-09-28

**Authors:** Ismaila Idowu Ahmed, Adeolu Adesoji Adediran, Raheem Abolore Yahya, Taiwo Yahaya, Segun Isaac Talabi, Jeleel Adekunle Adebisi, Rasheedat Modupe Mahamood, Jamiu Kolawole Odusote, Mariam Kehinde Sulaiman, Lawrence Aderemi Olatunji, Sulaiman Abdulkareem

**Affiliations:** a Department of Materials and Metallurgical Engineering, University of Ilorin, Ilorin, Nigeria; b Department of Parasitology, University of Ilorin, Ilorin, Nigeria; c Department of Anatomy, University of Ilorin, Ilorin, Nigeria; d Department of Mechanical Engineering, University of Ilorin, Ilorin, Nigeria; e Department of Mechanical Engineering, Landmark University, Omu-Aran, Nigeria; f Department of Metallurgical and Materials Engineering, Air Force Institute of Technology, Kaduna, Nigeria; g Department of Mechanical Engineering Science, University of Johannesburg, Johannesburg, South Africa

**Keywords:** Grinding disc, Corrosion, Potentiodynamic scan, Gasometry, Gastric solution, Food contamination

## Abstract

The need for food size reduction before consumption has led to the use of motorized grinding machine which operates on energized rubbing of two grooved cast-iron discs, and this unintentionally results in tribological degradation and corrosion of grinding discs into the ground food. The objective of this study was to carry out an assessment of corrosion susceptibility of grinding discs from different manufacturing methods in simulated gastro-intestinal environment. Six grinding discs from three states in Nigeria were selected for this study, based on manufacturing methods namely: rotary, cupola, and pit furnaces. Experimental techniques used for the study included: X-Ray Fluorescence spectroscope for determination of chemical composition and X-Ray Diffractometer was used for phase identification. Corrosion susceptibility of grinding discs on interaction with pseudo-body fluid was studied using potentiodynamic polarization scan and product analysis (gasometric) methods in simulated gastro-intestinal environment, typical of human stomach, as electrolyte. The electrolyte contained 2 g/L NaCl acidified to pH of 1.7 with HCl and regulated at 37 °C. Optical microscopy of the electrochemical samples was done for corrosion damage assessment. The key finding from the study was that all the grinding discs contain iron and silicon as dominant alloy elements, which existed predominantly as iron carbide and ferrosilicon phases. Corrosion of the discs in simulated gastric solution was well profound irrespective of the manufacturing method, though, with varying degree among the discs. The outcome of this study is applicable to food industries where cognitive measures may have to be taken on materials selection to minimise the risk of food contamination from materials corrosion.

## Highlights

• The investigated cast iron grinding discs exhibited profound corrosion susceptibility in simulated gastro-intestinal solution.
• There was no significant effect of grinding disc manufacturing methods on the corrosion degradation of the disc in gastric solution.
• Porosity (casting defect) observed in one of the discs aggravated severity of its corrosion degradation and thus necessitated quality control during production.

## 1 Introduction

Size reduction is a food processing technique often carried out before consumption particularly grains and vegetables. The traditional tools often used for the processing include stones [[1], [2], [3]], pestle and mortar [4], which are relatively effective but inefficient and time-consuming. The ever-increasing population further demanded faster and more efficient processing methods. This resulted in development of age long technology which operates on energized rubbing of two grooved cast-iron discs, that still dominates food grinding hitherto. The discs are largely produced from cast-iron scraps melted using different furnaces (Rotary, Pit and Cupola) operated by small scale foundry operators with little or no quality control measures. The operation of grinding discs in food grinding machine results in tribological degradation and corrosion of discs when in contact with water. It is quite disturbing to know from recent studies that up to 1.4 g of iron filings of about 12 nm grain size find their way into 1 kg of food processed using these grinding discs. Hence, it became imperative to assess the corrosion susceptibility of these discs materials when ingested into the human body system. Furthermore, persistent wearing and corrosion of the grinding discs during food processing may end up contaminating ground food with far reaching implications on health and safety of citizenry feeding on ground foods. Data from the World Health Organization affirms that approximately 10% of individuals fall ill globally from eating contaminated food thus leading to 420,000 deaths annually [5]. Hence, consumption of processed foods laden with metal chips and corroded particles, in most households which rely on grinding machine for processing staples grains (such as millets and sorghum) particularly in Africa and Asia, may constitute health risk that requires urgent attention.

The aim of the research was to conduct assessment of corrosion susceptibility of food grinding discs in gastro-intestinal environment, a bid to understand the chemical interaction of ingested disc particles with food into human alimentary canal system.

## 2 Materials and experimental methods

### 2.1 Materials

**Fig. 1:**
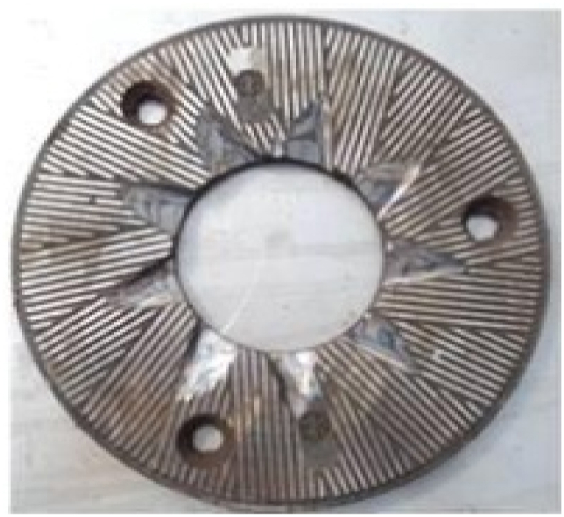
Grinding disc.

The cast-iron grinding discs (Fig. 1) used for the study were sourced, from two states in Nigeria (Osun and Kwara) and one imported from India, based on manufacturing methods namely: rotary, cupola and pit furnaces. Traditionally, food grinding discs are often produced from scrap cast-irons melted using different manufacturing methods above, with little or no quality control measures. A total of six samples used for the research and nomenclature adopted for each are shown in Table 1.

**Table 1:** Disc nomenclature and source location.

Disc Name	Production furnace	Source location
ROS	Rotary	Osun
RIL	Rotary	Kwara
RIW	Rotary	Osun
PIL	Pit	Kwara
CIW	Cupola	Osun
ID	N/A	India

### 2.2 Experimental methods

#### 2.2.1 Determination of chemical composition

Elemental chemical composition analysis was carried out on disc samples using XRF Mini Pal 4 version PW 4030 operated at 30 keV [6]. Samples subjected to metallographic sample preparation. X-ray produced by Cu anode incident on sample caused emission of a characteristic fluorescence x-ray from sample which is detected and measured with the aid of Mini Pal analytical software. The intensity of emitted fluorescence rays from the sample is proportional to the elemental concentration and recorded as spectra of intensity against peak position. Mini pal analytical software was used for data fitting and correlation with database for qualitative and quantitative evaluation of the elements in the sample.

#### 2.2.2 Phase identification with XRD

X ray diffraction was carried out on cast samples using Panalytical Empyrean X-ray Diffractometer for identification of metallurgical phases present in the samples. XRD data were collected with a copper (Ka-XRD-740) anode radiation tube. A divergence and scatter slit of 1.0° each and a receiving slit of 0.3 mm were used. The power rating of the X-ray generator used was 40 mA and 45 kV. Six samples from different manufacturing methods were examined at ambient temperature. X-ray intensities were measured at Bragg angle 2θ [7,8] between 15° and 80° and at a continuous scanning speed of 10°/min. A sampling pitch of 0.1° and the preset time of 0.6 s were used for all the samples.

#### 2.2.3 Potentiodynamic polarization scan and gasometry

Corrosion susceptibility of samples were tested using electrochemical methods of open circuit potential (OCP) and potentiodynamic polarization scan, and corrosion product analysis (gasometry) method. Potentiodynamic polarization scan was conducted in a 170 ml standard three-electrode electrochemical cell using Corrtest EIS Potentiostat/Galvanostat model CS150 installed with analytical software in line with ASTM G61 standard [9]. Each sample of 1 cm^2^ was spot welded with conducting wire and then mounted in cold resin, for electrical connection with Potentiostat. Samples were then subjected to metallographic sample preparation prior to polarization scan. The polished sample was connected to Potentiostat through the working electrodes terminal, while platinum wire was used as a counter electrode and saturated calomel electrode was used as reference electrode. The Simulated gastro-intestinal solution used as electrolyte contained 2 g/L of sodium chloride (NaCl) acidified to pH of 1.7 with hydrochloric acid (HCl) and maintained at temperature of 37 °C which is typical of the human alimentary canal system [10,11]. Open circuit potential (OCP) was carried out for 300 s and this is followed by potentiodynamic polarization scan at the rate of 0.2 mV/s between 0.1 to OCP and 0.3 V to OCP [12]. Subsequently, the electrochemical sample was rinsed in deionised water, acetone and then dried with hot air stream for optical microscopic assessment of corrosion degradation of the sample surface exposed to electrolyte.

The measurement of corrosion product by gasometric measurement of hydrogen gas evolution from samples immersed in a simulated solution over a given time was also carried out as supplementary method. Gasometric test was carried out to quickly assess corrosion susceptibility of the samples in simulated gastro-intestinal solution. The surface area of the samples was measured before insertion into the conical flask containing 100 ml of simulated solution for 120 min, gasometry was achieved from the volume of water displacement as a result of hydrogen gas evolution in line with the method and setup reported elsewhere [[13], [14], [15]].

## 3 Results and discussion

### 3.1 Chemical composition analysis with XRF

The results of X-ray Fluorescence spectroscopy are shown in Table 2. XRF results give a more reliable and detailed quantitative assessment of elemental composition of the disc samples. The results showed iron as the dominant element in their alloy composition. The exceptionally low Fe composition of 65.169 and 57.423 wt% in RIL and CIW sample respectively could be attributed to high amount of oxygen probably from oxides which amount to 29.593 and 23.617 wt% respectively.

**Table 2:** Elemental chemical composition (wt%).

Element	O	Mg	Al	Si	S	Cl	Ca	Cr	Mn	Fe	Co	Cu	Sn
**ROS**	0.00	0.65	3.53	4.22	0.15	0.39	0.25	0.17	0.59	88.86	0.29	0.15	0.00
**RIL**	29.59	0.02	1.92	0.96	0.06	0.00	0.85	0.00	0.03	65.17	0.04	0.05	0.52
**RIW**	0.00	0.00	2.98	2.09	0.33	0.32	0.61	0.13	0.07	92.52	0.26	0.04	0.05
**PIL**	0.00	0.00	2.30	1.47	0.00	0.26	0.68	0.11	0.26	92.97	0.22	0.23	0.06
**CIW**	23.62	2.29	1.91	3.80	0.66	2.50	4.34	0.01	0.08	57.42	0.01	0.04	2.03
**ID**	0.00	0.00	2.63	2.04	0.32	0.27	0.58	0.27	0.64	92.08	0.19	0.12	0.31

The rest four samples (ROS, RIW, PIL and ID) contain Fe composition which ranges between 88.855 and 92.966 wt%. Aluminum and Silicon alloy elements were also observed to be present in significant proportion. The elemental chemical composition is characteristic of cast-iron particularly the ROS, RIW, PIL and ID.

### 3.2 Result of X-ray diffraction

**Fig. 2:**
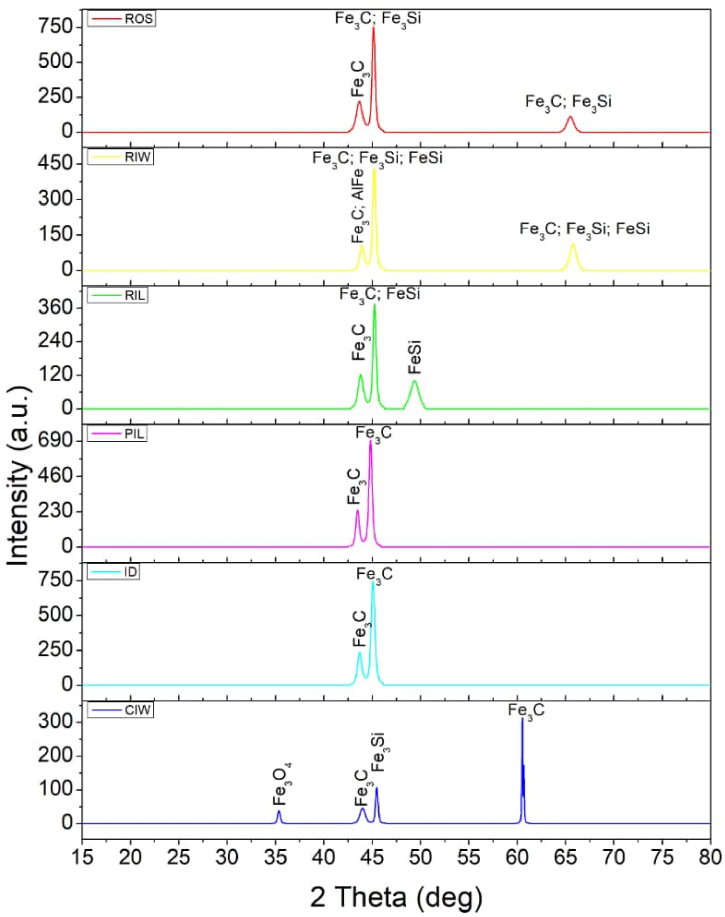
X-ray diffractometry of grinding disc samples.

The results of XRD are shown in Fig. 2 containing diffraction spectra of disc samples. All the six samples revealed the presence of cementite phase referred to as iron carbide (Fe_3_C) which is an equilibrium phase rich in iron and significant amount of carbon. This is an indication that all the discs are cast-iron. Fig. 2 also revealed the presence of ferroalloys particularly ferrosilicon (FeSi) which was observed in four of the samples tested except PIL and ID. There was also spectrum of magnetite (Fe_3_O_4_) observed in CIW sample. This observation is consistent with the XRF result which showed significant amount of oxide in CIW sample.

### 3.3 Corrosion test

The grinding disc is made from cast iron and are quite susceptible to corrosion. The results of the two supplementary tests carried to assess corrosion susceptibility of the disc were electrochemical techniques comprising of open circuit potential and potentiodynamic polarization scan, and lastly gasometric study of the corrosion product.

#### 3.3.1 Open circuit potential

**Fig. 3:**
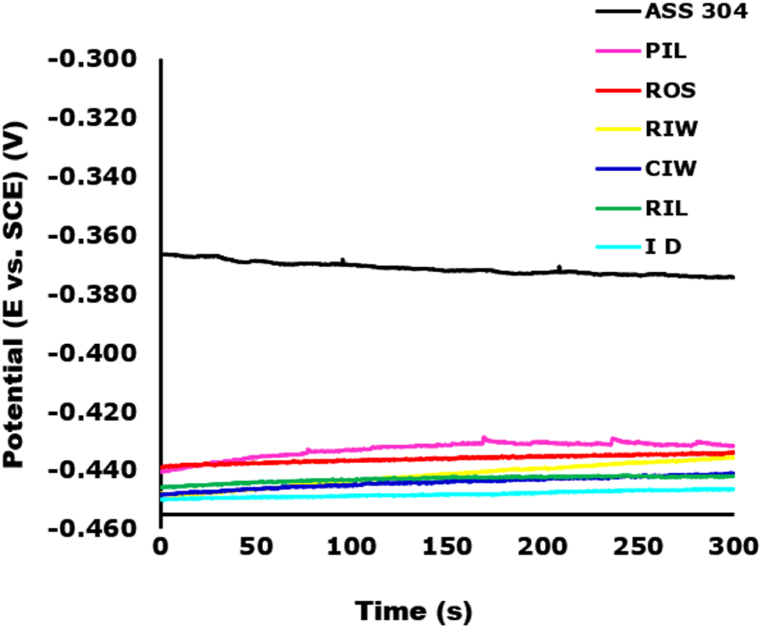
Result of open circuit potential of disc in simulated gastro-intestinal solution.

The results of open circuit potential of all the discs are shown in Fig. 3 as a plot of potential vs reference electrode (saturated calomel electrode) against time. The results showed the order of nobility of the tested discs based on their respective OCP and then benchmarked with austenitic stainless steel; type 304 (ASS 304) widely used globally for food processing. Expectedly, ASS 304 appeared as the most noble with OCP of −0.374 V after 300s. The order of nobility of all the disc tested including stainless steel, was ASS > PIL > ROS > RIW > CIW > RIL > ID. Among the cast iron disc tested, PIL and ID disc was ranked highest and lowest in order of nobility with OCP of −0.432 and −0.446 V respectively. The order nobility suggests that PIL is likely to be more resistant to corrosion susceptibility and ID appeared to be most vulnerable to corrosion degradation into the ground food. However, there was no any significant indication of the effect of disc manufacturing method on the observed results.

#### 3.3.2 Potentiodynamic polarization scan

**Fig. 4:**
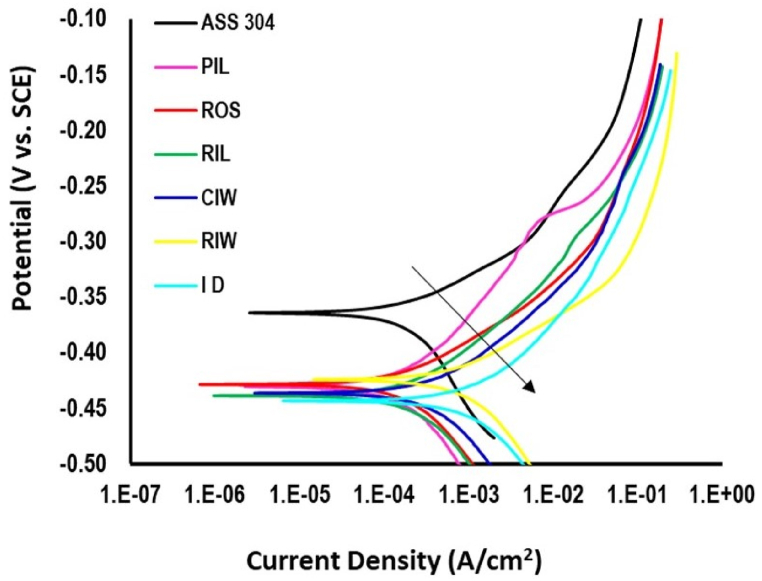
Potentiodynamic polarization scan of ASS 304 and cast-iron samples.

The results of potentiodynamic polarization scan carried out on disc sample is shown in Fig. 4 as plot of electrode potential versus saturated calomel electrode against corrosion current density. The graph showed cathodic and anodic portion of the corrosion reaction, wherein the sample is protected cathodically and where it began to corrode respectively. The corrosion potential (Eo) and corrosion current density (io) were obtainable from Tafel slope and analyses of polarization curve (see Table 3). Similarly, ASS 304 used as benchmark has corrosion potential of - 0.36387 V which is significantly higher that cast-irons samples. ID sample was observed to have the least nobility with corrosion potential of −0.44288 V. The order of nobility of the overall samples based corrosion potential, Eo was ASS > RIW > ROS > PIL > CIW > RIL > ID. The ranking of samples in order of corrosion resistance based on corresponding corrosion current density, io obtained from analyses of the polarization curve, was ASS > PIL > ROS > RIL > CIW > RIW > ID. Austenitic stainless steel has least rate of corrosion (2.57 mm/a) and ID samples consistently came up with highest rate of corrosion (21.95 mm/a). The corrosion parameters of other samples are available in Table 3. This is a region that characterise the localised corrosion where corrosion rate is more potent probably due to presence of aggressive corrosion species including chloride ion present in hydrochloric acid used for acidification of the simulated gastro-intestinal solution used for the test.

**Table 3:** Polarization curve analysis.

Sample	βa (mV)	βc (mV)	Corrosion current density, io (A/cm2)	Eo (V)	Corrosion rate (mm/a)	Residual
**ASS**	46.524	129.07	0.000223	−0.36387	2.5765	0.00000096
PIL	99.142	130.15	0.000247	−0.43011	3.1641	0.00300640
ROS	63.182	114.74	0.000265	−0.42863	3.3956	0.00582730
RIL	80.453	123.38	0.000341	−0.43735	4.7038	0.00073126
CIW	76.076	125.99	0.000595	−0.43609	7.6325	0.00183900
RIW	53.847	100.71	0.000954	−0.42439	12.243	0.00063336
ID	91.721	123.56	0.001710	−0.44288	21.95	0.00031917

In the case of food grinding disc, the most probable cause of corrosion could therefore be attributed to galvanic contact between dissimilar metallic materials usually between steel shaft and cast-iron disc used for grinding. The tendency for corrosion resulting from galvanic contact between the pair of discs used for grinding is quite low based on finding from this study. The manufacturing method used for disc production does not appear to have any significant effect on the corrosion susceptibility of the selected disc used for this study, due to marginal difference between the various disc potentials compared to the ASS. This may provide justification for reddish coloration usually observed few hours after wet grinding operation.

#### 3.3.3 Post-corrosion optical microscopy

**Fig. 5:**
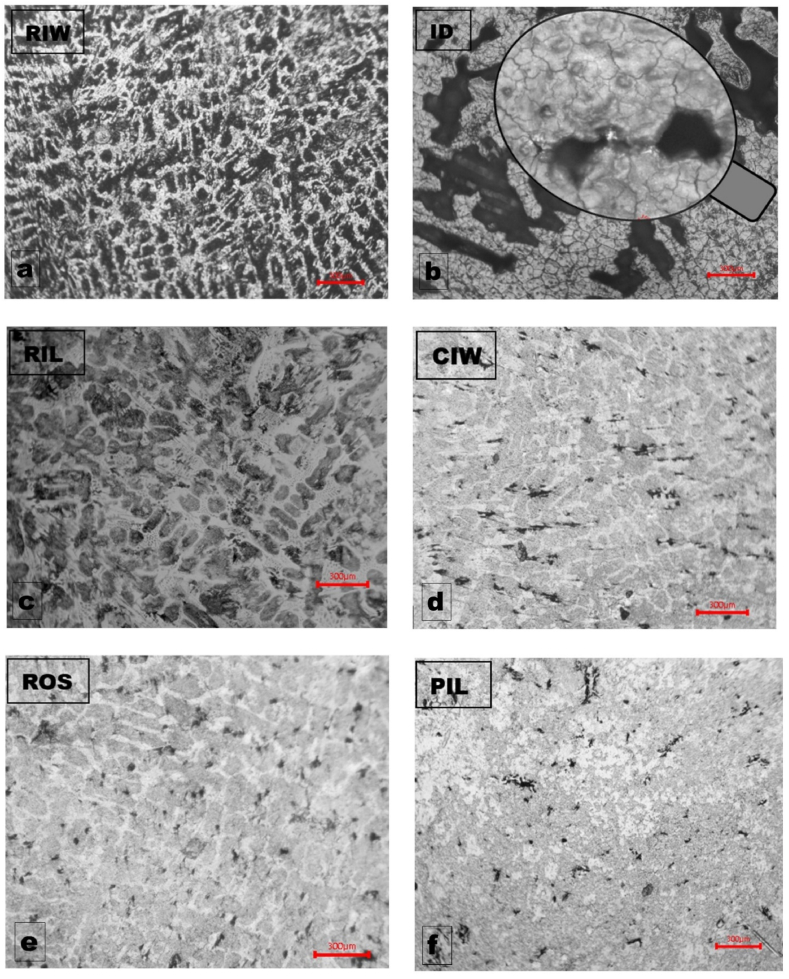
Optical micrographs of cast-iron discs after electrochemical potentiodynamic polarization scan showing disc sample (a) RIW, (b) ID, (c) RIL, (d) CIW, (e) ROS and (f) PIL.

The optical micrographs of all the discs subjected to electrochemical potentiodynamic polarization scan are shown in Fig. 5(a–f). The micrographs revealed the extent of corrosion following anodic polarization of the samples. There was evidence of severe corrosion of the sample, particularly RIW sample (Fig. 5a) which appeared to be most susceptible.

One dominant microstructural phase observed with XRD was cementite, which is an equilibrium microstructure in addition to pearlite (an interlamellar layers of ferrite and cementite microconstituents). This explains why cementite was the dominant phase observed by XRD and visible in the RIW micrograph as white background (Fig. 5a). Cementite phase is supersaturated with carbon, thus is relatively harder than ferrite. However, the ID micrograph (Fig. 5b) is also badly corroded next to the RIW. The ID micrograph also showed evidence of intergranular cracks and porosity as shown in inset on Fig. 5b. The intergranular cracks may be attributed to hydrogen embrittlement due to hydrogen gas evolution caused by displacement reaction between the iron and chloride as shown in Equation (1) in line the results of the gasometric studies. The porosities observed in the ID micrographs could be attributed to the casting defect and may have aggravated the extend of ID susceptibility to corrosion either as potential sites for pitting and/or crevice corrosion. The microstructures in disc samples RIL, CIW and ROS (Fig. 5 c, d and e respectively) are similar except the severity of corrosion. The PIL (Fig. 5f) observed to be most noble from polarization scan also showed least severity of corrosion.

(1) $$Fe+2HCl\;\to Fe{Cl}_{2}+H_{2}↑$$

**Fig. 6:**
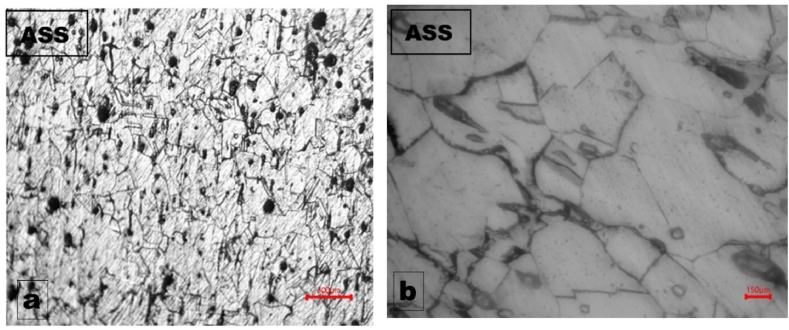
Optical micrographs of ASS 304 sample after electrochemical potentiodynamic polarization scan at (a) low magnification and (b) higher magnification.

The corrosion result of the grinding disc was benchmarked with austenitic stainless steel, type 304 (ASS 304) and was observed as the most noble from polarization scan results. The optical micrographs of the ASS 304 at low and higher magnifications (Fig. 6 a and b respectively) showed little or no corrosion susceptibility which attests to corrosion superiority of stainless steels compared to cast-irons. This is not unexpected of stainless steels, because of their propensity for development of chromium oxide passive film that helps to protect their exposure to corrosion environment and slow down the corrosion rate [[16], [17], [18], [19]].

#### 3.3.4 Gasometric study

**Fig. 7:**
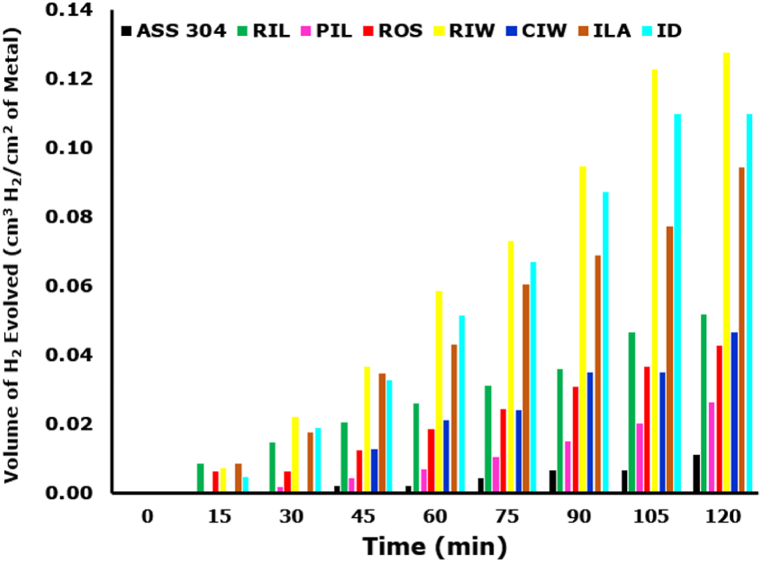
Gasometric study of discs in simulated gastro-intestinal solution at regular interval.

The results of gasometric tests are shown in Fig. 7 as a plot of volume of hydrogen gas produced as a product of corrosion against time. The hydrogen evolution occurred due to chemical reaction, according to Equation (1), between iron in the disc materials and hydrochloric acidic in gastric solution, which caused measurable water displacement. Initially, reaction commenced early in cast-iron discs at about 15 min, but no hydrogen gas evolution was observed from the ASS 304 samples exposed to the same gastric solution, until about 45 min. The early and consistently higher evolution of hydrogen gas from cast-iron discs is an indication of higher corrosion rate occurring on cast-iron discs compared to the stainless steel which is an alloy steel containing chromium greater than 12 wt%. Chromium is reputed for development of protective shield in corrosive environment through formation of tenacious chromium oxide film that protects the subcutaneous layer beneath the film from further exposure to aggressive environmental attacks [12,16,20].

**Fig. 8:**
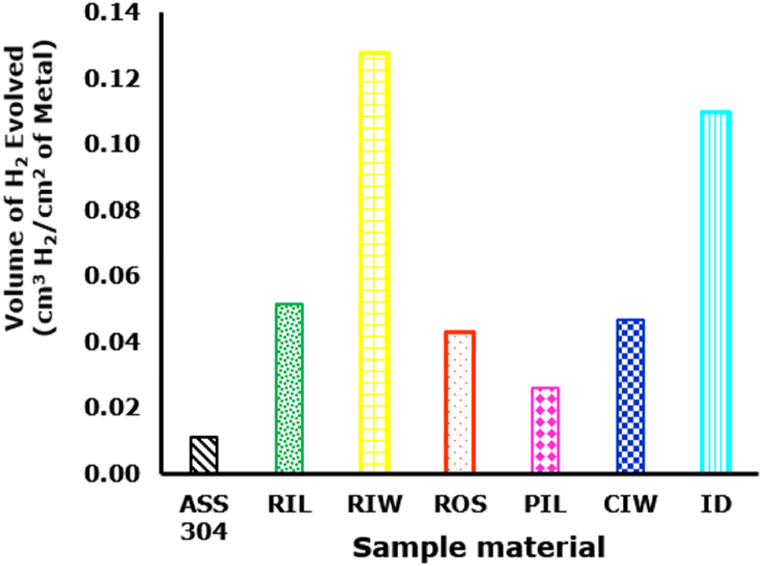
Gasometric study of grinding disc in simulated gastro-intestinal solution for 120 min.

The corrosion susceptibility among the discs is consistent at all times. While ASS 304 recorded the least hydrogen displacement on account of its corrosion superiority, CIW was consistently the most susceptible with highest hydrogen displacement. The plot of hydrogen evolution per surface area of sample against the disc type at immersion time of 120 min is shown in Fig. 8. The order of corrosion susceptibility is CIW > ID > RIL = CIW > ROS > PIL > ASS 304. The results of gasometric study are consistent with corrosion in potentiodynamic polarization scan of the discs when exposed simulated gastric solution for 300 s. Again, there was no observed correlation between manufacturing method and corrosion susceptibility.

## 4 Conclusions

The research on the assessment of corrosion susceptibility of the food grinding discs in gastro-intestinal environment has been carried out and the following are the conclusions reached at the end of the study:

1. Chemical composition analyses showed significant amount of iron, silicon and aluminum which appeared as iron carbide and ferroalloys of silicon and aluminum in nearly all samples tested is an indication that all discs tested are cast-irons.
2. Corrosion tests carried out on the disc by open circuit potential (OCP), potentiodynamic polarization scan and gasometry showed profound corrosion susceptibility of discs in gastric solution. However, there was no significant indication of the effect of manufacturing method on corrosion susceptibility of the discs tested.
3. The ranking in the order of nobility of the grinding discs based on the marginal difference in electrode potential showed RIW and ID as the most susceptible to corrosion degradation, while PIL was found to be more noble. The severity of corrosion susceptibility in ID was probably aggravated by casting defect and intergranular cracking.
4. Significant difference was observed on the corrosion nobility of austenitic stainless steel (ASS 304) compared to cast-iron disc, which suggested superiority of stainless steel to the cast-iron grinding discs in terms of corrosion resistance and suitability for food processing and handling.

## Data availability statement

No data was used for the research described in the article.

## Additional information

No additional information is available for this paper.

## CRediT authorship contribution statement

**Ismaila Idowu Ahmed:** Conceptualization, Investigation, Writing – original draft, Writing – review & editing, Data curation. **Adeolu Adesoji Adediran:** Resources, Writing – original draft, Writing – review & editing. **Raheem Abolore Yahya:** Investigation, Resources, Writing – review & editing, Data curation. **Taiwo Yahaya:** Data curation, Resources, Writing – review & editing, Formal analysis. **Segun Isaac Talabi:** Investigation, Resources, Writing – review & editing, Data curation. **Jeleel Adekunle Adebisi:** Data curation, Resources, Writing – review & editing, Formal analysis. **Rasheedat Modupe Mahamood:** Resources, Writing – review & editing. **Jamiu Kolawole Odusote:** Data curation, Resources, Writing – review & editing, Formal analysis. **Mariam Kehinde Sulaiman:** Investigation, Resources, Writing – review & editing, Data curation, Formal analysis. **Lawrence Aderemi Olatunji:** Investigation, Resources, Writing – review & editing, Data curation, Formal analysis. **Sulaiman Abdulkareem:** Conceptualization, Funding acquisition, Writing – original draft, Writing – review & editing.

## Declaration of competing interest

The authors declare that they have no known competing financial interests or personal relationships that could have appeared to influence the work reported in this paper.

## References

[1] Richard C.E., Ugochukwu G.M., Omaka N.O. (2010). Food grinding stones as a source of heavy metals contamination of diets. J. Sci. Multidiscip. Res..

[2] Anthony B. (2013). Some Nigerian traditional food milling techniques and cookware increase concentrations of some heavy metals in lycopersicon esculentum and Citrullus lanatus. IOSR J. Pharm..

[3] Ehiri R.C., Megwa U.G., Omaka O.N. (2010). Food grinding stones as a source of heavy metal contamination of diets. J. Sci. Multidiscip. Res..

[4] Nnaji J.C., Emmanuel P.N. (2016). Trace metals in food condiments processed with manual metallic grinders. Phamaceut. Chem. J..

[5] World Health Organization (2015). Disease outbreak news: cholera – United Republic of Tanzania. https://reliefweb.int/report/united-republic-tanzania/disease-outbreak-news-cholera-united-republic-tanzania.

[6] Jamok E.J. (2014). Radiological and X-ray fluorescence spectrometric analyses of selected inorganic fertilizers in zaria kaduna state, Nigeria: source of possible environmental pollution. Int. J. Sci. Res..

[7] Ahmed I.I. (2018). Investigation of surface residual stress profile on martensitic stainless steel weldment with X-ray diffraction. J. King Saud Univ. - Eng. Sci..

[8] Cullity B.D., Stock S.R. (2001).

[9] ASTM (2004). Designation: G5-94.

[10] Ghazal H. (2009).

[11] Mokarram R.R. (2009). The influence of multi stage alginate coating on survivability of potential probiotic bacteria in simulated gastric and intestinal juice. Food Res. Int..

[12] Ahmed I.I. (2015). Stress corrosion cracking of austenitic stainless steels in potentiostatically controlled chloride environments at ambient temperature. Ann. Facul. Eng. Hunedoara - Int. J. Eng..

[13] Huang J., Fisher P.R., Argo W.R. (2007). A gasometric procedure to measure residual lime in container substrates. Hortscience.

[14] Obot I., Umoren S., Obi-Egbedi N. (2011). Corrosion inhibition and adsorption behaviour for aluminuim by extract of Aningeria robusta in HCl solution: synergistic effect of iodide ions. J. Mater. Environ. Sci..

[15] Sippel T.R., Pourpoint T.L., Son S.F. (2013). Combustion of nanoaluminum and water propellants: effect of equivalence ratio and safety/aging characterization. Propellants, Explos. Pyrotech..

[16] Ahmed I.I. (2017). Analysis of intergranular carbide precipitate in HAZ of martensitic stainless steel. J. Eng. Sci. Technol..

[17] Ahmed I.I. (2016). Microstructural correlation of hardness profile in martensitic stainless steel weldment. Metallogr. Microstruct. Anal..

[18] Cui J.-L. (2016). Electrochemical properties of tungsten-alloying-modified AISI 430 stainless steel as bipolar plates for PEMFCs used in marine environment. Acta Metall. Sin..

[19] Liu Y. (2021). Performance of Nb 0.8 Zr 0.2 layer-modified AISI430 stainless steel as bipolar plates for direct formic acid fuel cells. Acta Metall. Sin..

[20] Ahmed I.I. (2016). Assessment of crack propagation mode in martensitic stainless steel HAZ with electron back scatter diffraction: effects of environmental variables. Ann. Facul. Eng. Hunedoara - Int. J. Eng..

